# Genome-wide Analysis of the WRKY Gene Family and its Response to Abiotic Stress in Buckwheat (*Fagopyrum Tataricum*)

**DOI:** 10.1515/biol-2019-0010

**Published:** 2019-03-20

**Authors:** Xia He, Jing-jian Li, Yuan Chen, Jia-qi Yang, Xiao-yang Chen

**Affiliations:** 1 ushan road NO.483 Guangzhou city, Guangdong Guangzhou, P.R.China; 2 State Key Laboratory for Conservation and Utilization of Subtropical Agro-bioresources (South China Agricultural University), Guangzhou 510642, China; 3 Guangdong Key Laboratory for Innovative Development and Utilization of Forest Plant Germplasm, Guangzhou 510642, China; 4 Guangdong Province Research Center of Woody Forage Engineering Technology, Guangzhou 510642, China; 5 College of Forestry and Landscape Architecture, South China Agricultural University, Guangzhou 510642, China

**Keywords:** *Fagopyrum tataricum*, WRKY gene family, bioinformatic, genome-wide analysis, abiotic stress

## Abstract

The WRKY gene family is an ancient plant transcription factor (TF) family with a vital role in plant growth and development, especially in response to biotic and abiotic stresses. Although many researchers have studied WRKY TFs in numerous plant species, little is known of them in Tartary buckwheat (*Fagopyrum tataricum*). Based on the recently reported genome sequence of Tartary buckwheat, we identified 78 FtWRKY proteins that could be classified into three major groups. All 77 WRKY genes were distributed unevenly across all eight chromosomes. Exon–intron analysis and motif composition prediction revealed the complexity and diversity of FtWRKYs, indicating that WRKY TFs may be of significance in plant growth regulation and stress response. Two separate pairs of tandem duplication genes were found, but no segmental duplications were identified. Overall, most orthologous gene-pairs between Tartary and common buckwheat evolved under strong purifying selection. qRT-PCR was used to analyze differences in expression among four FtWRKYs (FtWRKY6, 74, 31, and 7) under salt, drought, cold, and heat treatments. The results revealed that all four proteins are related to abiotic stress responses, although they exhibited various expression patterns. In particular, the relative expression levels of FtWRKY6, 74, and 31 were significantly upregulated under salt stress, while the highest expression of FtWRKY7 was observed from heat treatment. This study provides comprehensive insights into the WRKY gene family in Tartary buckwheat, and can support the screening of additional candidate genes for further functional characterization of WRKYs under various stresses.

## 1 Introduction

Although most plants grow in specific environments, they experience continual changes in their external conditions, therefore, plants have developed a series of complex mechanisms to withstand stresses [1, 2]. Transcription factors (TFs) are crucial proteins in the response to environmental stimuli by regulating gene expression temporally and spatially [3]. TFs, also called sequence-specific DNA-binding factors, bind to conserved cis-elements in promoter regions, thereby interacting with downstream target genes to influence transcription [4, 5]. The WRKY family is one of the largest and most diverse TF families, and is related to the coordination of many physiological activities in the plant kingdom. Moreover, it has been widely studied for its important role in regulating gene expression under adverse conditions [6, 7, 8, 9]. According to the number of domains and features of the zinc-finger motif, the WRKY family can be divided into three distinct groups (I, II, and III) [10]. Groups I and II both exhibit a C2H2 zinc-finger motif, although group I has two WRKY domains whereas group II has only one. Group II can be further classified into five subgroups (IIa–e) based on the amino acid (aa) stretch in the zincfinger motif [11, 12]. Group III also contains a single WRKY domain but a C2HC zinc-finger motif [11, 12]. The domain of WRKY TFs is approximately 60-aa long at the N-terminus and has a typical zinc-finger motif at the C-terminus [13]. It is generally thought that WRKY TFs can specifically interact with the W-box (TTGACT/C) found in the promoter region of many plants [14, 15]. The first WRKY gene, SPF1, was isolated from sweet potato (*Ipomoea batatas*), and was considered to have potential negative impacts on the regulation of sucrose-induced genes [16]. Multiple studies have since been performed in different plants. For example, 72 and 64 WRKYs have been reported in the model herbaceous plants *Arabidopsis thaliana* and *Oryza sativa*, respectively [17], and many other studies (Table S1) have been performed in *Triticum aestivum* [18], *Camellia sinensis* [19], *Populus trichocarpa* [10], and *Glycine max* [20]. Such studies have demonstrated that WRKY proteins not only participate in physiological processes such as seed germination [21] and leaf senescence [22], but are also involved in the response to biotic stresses such as pathogens [23] and pests [24], as well as abiotic stresses such as drought [25], heat [26], cold [27], salinity [28], and heavy metals [8]. For instance, in transgenic tobacco, the BcWRKY46 gene enhanced tolerance to freezing, abscisic acid, salt, and dehydration stresses [29]. Meanwhile, ThWRKY7 improved cadmium tolerance under cadmium stress in combination with ThVHAc1 in woody plants [30]. In *A. thaliana*, AtWRKY25, 33, 46, and 54 have been demonstrated to play vital roles in the response to several types of stress [31, 32]. Moreover, WRKY proteins have vital roles in the biosynthesis of secondary metabolites, such as paclitaxel and benzylisoquinoline [33, 34].

Tartary buckwheat (*Fagopyrum tatarium*) is an annual eudicot plant belonging to the genus *Fagopyrum*. Tartary buckwheat and common buckwheat (*Fagopyrum esculentum*) are the most commonly cultivated species of this genus [35, 36]. Tartary buckwheat originates in southwest China, and is also known as kuqiao for its bitter seeds. It is grown mainly in farming and ranching areas that overlap with northern China, and exhibits strong abiotic resistance to harsh eco-climatic environments [37, 38, 39]. As a medicinal and nutrient-rich crop, Tartary buckwheat has higher flavonoid content than common buckwheat and is especially abundant in rutin, accounting for 0.8–1.7% of the dry weight of the plant [40, 41]. Moreover, quercetin, anthocyanins, and other flavonoids in buckwheat have various biological activities, such as antibacterial, antioxidant, and anti-inflammatory effects [41, 42, 43, 44].

The genome sequence of Tartary buckwheat was recently published [42], enabling the systematic characterization of WRKY genes in this species and the study of their expression. Therefore, we performed bioinformatics analyses including phylogenetic, gene structure, and motif composition analyses; determined the chromosomal locations of the genes; and calculated the Ka/Ks values of WRKY genes in Tartary buckwheat. Subsequently, the expression patterns of select FtWRKYs under salt, drought, cold, and heat treatments were analyzed. This study helps clarify the functions of WRKY proteins and provides a foundation for further comparative genomic studies in Tartary buckwheat.

## 2 Materials and Methods

### 2.1 Identification of putative WRKY proteins in Tartary buckwheat

To accurately identify WRKY TFs in Tartary buckwheat, we downloaded the whole genome sequence from the MBKbase website (http://www.mbkbase.org/Pinku1) [42]. Moreover, the Hidden Markov Model profile of the WRKY domain (PF03106) was downloaded from the Pfam family database (http://pfam.xfam.org/search) [45]. All possible WRKY proteins were searched using HMMER 3.0 (http://hmmer.janelia.org/) with the default parameters. In addition, we used both HMMER (http://plants.ensembl.org/hmmer/index.html) and SMART (http://smart.embl-heidelberg.de/) to ascertain the presence of the WRKY domain [46]. Sequences with different domains or redundancies were excluded. AtWRKY data were obtained from the *Arabidopsis* genome TAIR website (http://www.Arabidopsis.org/index.jsp) [47].

### 2.2 Phylogenetic analysis and protein properties of FtWRKYs

Multiple alignment of the WRKY domain sequence in the 78 FtWRKY proteins was performed with ClustalW using the default settings [48], and a phylogenetic tree was constructed using the neighbor-joining (NJ) method with MEGA 6.0 software [49] with the following parameters: pairwise deletion, 1000 bootstrap replicates, and Poisson correction. To obtain an accurate classification, two members per group and highly conserved representative AtWRKY proteins were included in the tree building (Table S2). The FtWRKY domains in each group were analyzed, and the sequence logos were produced using WebLogo online software (http://weblogo.threeplusone.com/) [8]. The properties of the proteins, including sequence length (aa length), molecular weight (MW), isoelectric point (IP), instability index (II), aliphatic index (AI), and grand average of hydropathicity (GRAVY) were calculated using the ExPASy website (http://web.expasy.org/protparam/), while the subcellular localization of each protein was predicted with Cell-PLoc [50].

### 2.3 Conserved motifs and gene structure analysis of FtWRKY genes

The conserved motifs in the FtWRKY proteins were predicted using MEME Suite (http://meme-suite.org/tools/meme), the following parameters were employed in analysis: maximum number of motifs 10; minimum motif width 6; maximum motif width 50. The gene structure of FtWRKY was predicted by comparing the coding sequences and corresponding genomic sequences using the GSDS tool (http://gsds.cbi.pku.edu.cn) [51].

### 2.4 Chromosomal location of FtWRKY genes

The physical location of FtWRKYs on chromosomes was retrieved from the annotated genome and chromosome files, and genes were plotted separately onto all eight chromosomes based on the order of their physical position. Finally, an image of their physical location was created with MapInspect software [52].

### 2.5 Calculation of Ka/Ks of orthologous gene-pairs between FtWRKYs and FeWRKYs

The orthologous gene-pairs of WRKY between Tartary and common buckwheat were aligned using ClustalW on the basis of diverse sequence alignment tools. Alignment of the aa sequences and their corresponding original cDNA sequences were used to calculate the synonymous rate (Ks) and nonsynonymous rate (Ka) using the CODEML program in the PAML interface tool of PAL2NAL [5]. Furthermore, the evolutionary constraint (Ka/Ks) was determined. The approximate time (million years ago [Mya]) of the orthologous gene-pairs were estimated using the equation T = Ks/2λ, where the synonymous substitution rate (λ) was 1.5 × 10^-8^ [53].

### 2.6 RNA-sequencing data analysis of FtWRKY genes

To investigate the expression patterns of FtWRKYs among different tissues as well as under salt treatment, the Illumina RNA-sequencing datasets were collected from the NCBI SRA database (https://www.ncbi.nlm.nih.gov/sra), including different five tissues, i.e. roots, flowers, stems, and leaves (accession: SRX3974871; SRX3974872; SRX3974873; SRX3974874), and for the salt treatment, plants were disposed with 200 mM NaCl for 0, 24 hours (accession:SRX3210945; SRX3210946). Transcript expression levels were calculated in FPKM units as reads per kilobase of transcript sequence per million mapped reads. FPKM value were transformed by log2 and the heatmap was performed by HemI software [54].

### 2.7 Plant material, growth conditions, and treatments

The seeds of Tartary buckwheat cv. Pinku1 were provided by Dr. Bo Li from the college of agriculture, South China Agricultural University. Plants were grown in pots containing soil and vermiculite mixture (3:1) in an artificial climate chamber, with a program set to 25/22°C (day/night), 16-h photoperiod, and relative humidity of 75%. Stress treatments were initiated in 5-week-old normal seedlings, and the seedlings were disposed with following treatment as described by Zhou *et al*. [38] and Gao *et al*. [40]. For salt and drought treatments, seedlings were irrigated with 15% NaCl and 30% PEG6000, respectively. For cold and heat treatments, seedlings were transferred to 4°C and 40°C in an illuminated incubator, respectively. Whole samples were harvested after 0, 3, and 12 h, while plants at 0 h were used as the control. Samples from three biological replicates were frozen immediately in liquid nitrogen and stored at -80°C until further analysis.

### 2.8 Total RNA extraction and cDNA reverse transcription

Total RNA was isolated from frozen seedlings with an RNAprep Pure Plant Kit (TIANGEN, Beijing, China) and the concentration of RNA was determined using spectrophotometry. First-strand cDNA was reverse transcribed using a PrimeScript RT Master Mix (Perfect) Real Time Kit (TaKaRa, Dalian, China). All operational procedures were performed according to the manufacturer’s instructions. Finally, 1 μL cDNA was diluted with 4 μL nuclease-free water before quantitative real-time (qRT)-PCR analysis.

### 2.9 qRT-PCR analysis

The expression of FtWRKY6 and 74 were enhanced significantly after treatment with NaCl, and same as FtWRKY7 and 31, they shared a high identity with AtWRKY25, 33, 46, and 54 were identified in plant defense experiments, thus were selected for qRT-PCR analysis. The housekeeping gene histone3 (GenBank ID: HM628903) of Tartary buckwheat was used as an internal control [55]. Specific qRT-PCR primers (Table S3) were designed with Primer Premier 6.0 and synthesized by Sangon (Guangzhou, China). The SYBR Premix Ex Taq Kit (TaKaRa, China) was used for the qRT-PCR reaction, and the reactions were performed using a Roche LightCyler 480 system (Roche, Basel, Switzerland). The qRT-PCR reactions were performed in a total volume of 20 μL, including 10 μL SYBR Premix Ex Taq, 6 μL ddH_2_O, 2 μL diluted cDNA, and 1 μL each forward and reverse primer. The qPCR program was as follows: initiation with a 3 min denaturation period at 95°C, followed by 40 cycles of 95°C for 15 s, 60°C for 30 s, and 72°C for 20 s. Three biological replicates and three technical replicates were included in the qRT-PCR analysis. Finally, gene expression was calculated using the 2^-ΔΔ^*c* method [56], and the means and standard deviation of three biological replicates were calculated. The significance was statistically analyzed by *t* text, and revealed by asterisks (* *p*<0.05, **p<0.01).

## 3 Results

### 3.1 Identification and classification of FtWRKYs

In the *F. tatarium* genome sequence, 74 protein sequences were detected that contained the conserved complete WRKY domain based on the HMMER and SMART analyses. Moreover, four additional proteins were identified that were missing the zinc-finger motif. These sequences were included for the subsequent analysis because they have been identified as annotated WRKYs in *Arabidopsis* based on BLAST, and this phenomenon has also been reported in *Capsicum annuum* [57]. Interestingly, one gene (FtPinG0001732900.01) showed two alternative messenger RNA splicing, therefore, 77 genes were detected in total. The 78 identified proteins were named FtWRKY1–77 according to the distribution (from top to bottom) of the corresponding genes on chromosomes (Chr.) 1–8 (table 1).

**Table 1:** Description of the WRKY proteins in Tartary buckwheat.

Protein name	Gene ID	Accession number	Group	Chr.	Protein length(aa)	Molecular weight(Da)	Theoretical IP	Aliphatic index	Grand average of hydropathicity	Instability index	subcellular localization
FtWRKYl	FtPinG0000171800.01	MK161300	Ile	Chr.l	379	41987.38	5.28	65.78	-0.686	54.04	Nucleus
FtWRKY2	FtPinG0000299100.01	MK161305	1	Chr.l	582	62963	6.45	66.36	-0.649	51.36	Nucleus
FtWRKY3	FtPinG0001428200.01	MK161291	1	Chr.l	153	17133.75	5.96	46.41	-1.166	51.73	Nucleus
FtWRKY4	FtPinG0002493300.01	MK161272	Ile	Chr.l	272	29546.32	5.7	56.32	-0.7	55.51	Nucleus
FtWRKY5	FtPinG0003193100.01	MK161301	lld	Chr.l	144	15924.29	9.53	43.33	-0.655	53.4	Nucleus
FtWRKYô	FtPinG0001577500.01	MK161279	1	Chr.l	523	57475.12	6.58	55.18	-0.852	58.96	Nucleus
FtWRKY7	FtPinG0004829100.01	MK161280	III	Chr.l	335	37272.07	6.82	51.85	-0.848	73.87	Nucleus
FtWRKY8	FtPinG0001878200.01	MK161333	lld	Chr.l	334	36276.22	9.82	67.72	-0.519	45.02	Nucleus
FtWRKY9	FtPinG0005486000.01	MK161313	Ile	Chr.l	172	19952.73	9.3	62.21	-0.843	58.86	Nucleus
FtWRKYlO	FtPinG0006017000.01	MK161273	Ile	Chr.l	293	32346.18	5.36	67.2	-0.619	59.74	Nucleus
FtWRKYll	FtPinG0001921200.01	MK161289	lld	Chr.l	263	29160.81	9.62	62.28	-0.807	59.99	Nucleus
FtWRKY12	FtPinG0003025100.01	MK161335	III	Chr.l	360	40227.07	6.07	66.92	-0.517	52.83	Nucleus
FtWRKY13	FtPinG0004319300.01	MK161332	lld	Chr.l	175	20144.18	9.88	49.54	-0.894	57.16	Nucleus
FtWRKY14	FtPinG0008726200.01	MK161345	Ile	Chr.2	274	30477.75	9.04	58.32	-0.897	48.22	Nucleus
FtWRKY15	FtPinG0005008400.01	MK161327	Ile	Chr.2	178	20159.06	6.21	44.33	-0.938	42.88	Nucleus
FtWRKYlô	FtPinG0005548400.01	MK161299	Ma	Chr.2	321	35837.17	6.96	67.17	-0.711	54.25	Nucleus
FtWRKY17	FtPinG0006962300.01	MK161326	Ile	Chr.2	248	28409.05	6.72	64.84	-0.85	53.55	Nucleus
FtWRKY18	FtPinG0007313500.01	MK161312	lld	Chr.2	294	33406.33	9.54	68.3	-0.709	41.77	Nucleus
FtWRKY19	FtPinG0004914600.01	MK161339	1	Chr.2	639	69367.94	5.91	55.85	-0.737	52.69	Nucleus
FtWRKY20	FtPinGOOO3OO13OO.Ol	MK161309	lld	Chr.2	327	35664.56	9.88	62.1	-0.599	53.04	Nucleus
FtWRKY21	FtPinG0003701200.01	MK161287	1	Chr.2	476	52191.55	9.23	58.17	-0.871	48.96	Nucleus
FtWRKY22	FtPinG0003722700.01	MK161306	lld	Chr.2	330	36055.55	9.62	63.24	-0.641	56.15	Nucleus
FtWRKY23	FtPinG0003761600.01	MK161334	Mb	Chr.2	537	57589.63	6.41	62.42	-0.683	53.67	Nucleus
FtWRKY24	FtPinG0006884300.01	MK161274	1	Chr.2	480	52906.17	5.72	63.56	-0.617	46.41	Nucleus
FtWRKY25	FtPinG0003815100.01	MK161283	1	Chr.2	319	35182.41	8.84	67.43	-0.784	61.55	Nucleus
FtWRKY26	FtPinG0004814300.01	MK161318	Ile	Chr.3	289	32264.84	6.66	55.95	-0.922	41.54	Nucleus
FtWRKY27	FtPinG0006083600.01	MK161319	Ile	Chr.3	304	33816.57	8.63	61.18	-0.687	50.98	Nucleus
FtWRKY28	FtPinG0007458900.01	MK161275	Ile	Chr.3	291	31784.28	6.44	67.01	-0.629	53.33	Nucleus
FtWRKY29	FtPinG0005089800.01	MK161338	Ile	Chr.3	228	26122.18	8.22	49.91	-0.86	50.3	Nucleus
FtWRKY30	FtPinG0005089400.01	MK161298	Ile	Chr.3	195	21966.61	9.05	64.9	-0.808	38.5	Nucleus
FtWRKY31	FtPinG0006388000.01	MK161325	III	Chr.3	278	31217.17	5.97	47.41	-0.879	55.09	Nucleus
FtWRKY32	FtPinG0000403800.01	MK161322	Ile	Chr.3	286	31863 .57	6.66	60.28	-0.771	55 .06	Nucleus
FtWRKY33.1	FtPinGOOO17329OO.Ol	MK161323	lld	Chr.3	155	17303 .72	10.04	53.48	-0.87	47 .98	Nucleus
FtWRKY33.2	FtPinGOOO17329OO.Ol	MK161323	lld	Chr.3	363	40614 .9	9.85	65.84	-0.804	55 .88	Nucleus
FtWRKY34	FtPinG0004464200.01	MK161285	Ma	Chr.3	304	34327 .33	6.16	69.9	-0.763	55	Nucleus
FtWRKY35	FtPinG0004460400.01	MK161337	Mb	Chr.3	538	57612 .46	8.9	57.47	-0.706	44 .3	Nucleus
FtWRKY36	FtPinG0002374400.01	MK161331	Ile	Chr.3	148	16780 .86	5.13	61.08	-0.81	56 .55	Nucleus
FtWRKY37	FtPinG0006311800.01	MK161284	Mb	Chr.3	359	37811 .64	9.35	57.66	-0.522	41 .67	Nucleus
FtWRKY38	FtPinG0008419400.01	MK161311	Ile	Chr.4	189	21067 .67	9.23	61.27	-0.807	38 .41	Nucleus
FtWRKY39	FtPinG0007629700.01	MK161271	III	Chr.4	342	38260 .19	4.97	54.53	-0.753	55 .17	Nucleus
FtWRKY40	FtPinGOOO51117OO.Ol	MK161316	1	Chr.4	494	53873 .54	7.09	54.88	-0.791	62 .48	Nucleus
FtWRKY41	FtPinGOOO51O55OO.Ol	MK161310	Mb	Chr.4	578	63106 .91	5.16	60.45	-0.744	48 .21	Nucleus
FtWRKY42	FtPinG0006617300.01	MK161320	1	Chr.4	490	53787 .68	7	55.12	-0.81	65 .75	Nucleus
FtWRKY43	FtPinG0000497200.01	MK161304	Ile	Chr.5	171	19859 .8	8.97	42.75	-1.187	44 .96	Nucleus
FtWRKY44	FtPinGOOO25245OO.Ol	MK161295	III	Chr.5	321	36241 .7	5.24	71.31	-0.585	49 .24	Nucleus
FtWRKY45	FtPinG0004671500.01	MK161329	1	Chr.5	548	60094 .97	5.92	67.08	-0.635	47 .41	Nucleus
FtWRKY46	FtPinG0004682800.01	MK161290	1	Chr.5	459	49709 .24	6.58	55.05	-0.763	65 .13	Nucleus
FtWRKY47	FtPinGOOO15326OO.Ol	MK161293	Mb	Chr.5	368	39517 .67	9.69	49.92	-0.649	55 .13	Nucleus
FtWRKY48	FtPinG0004237400.01	MK161342	Ma	Chr.5	163	18194 .55	6.22	78.28	-0.528	45 .55	Nucleus
FtWRKY49	FtPinG0007732600.01	MK161315	Ile	Chr.5	176	20396 .98	9.41	56.36	-1.035	50 .62	Nucleus
FtWRKY50	FtPinGOOO62O23OO.Ol	MK161286	Mb	Chr.5	246	26678 .17	8.25	61.63	-0.408	50 .03	Nucleus
FtWRKY51	FtPinGOOO62O13OO.Ol	MK161282	Mb	Chr.5	575	62401 .23	6.66	61.63	-0.688	43 .59	Nucleus
FtWRKY52	FtPinG0002688700.01	MK161277	Ile	Chr.6	230	26476 .19	6.12	55.04	-1.112	39 .34	Nucleus
FtWRKY53	FtPinG0002710400.01	MK161307	lld	Chr.6	319	34893 .69	9.58	64.73	-0.559	44 .81	Nucleus
FtWRKY54	FtPinG0007768300.01	MK161281	Ile	Chr.6	198	22167 .05	9.27	60.05	-0.868	38 .85	Nucleus
FtWRKY55	FtPinGOOO63223OO.Ol	MK161346	Ile	Chr.6	301	34764 .78	6.22	72.19	-0.664	71 .23	Nucleus
FtWRKY56	FtPinG0008708000.01	MK161347	Ma	Chr.6	379	41222 .95	8.89	67.18	-0.642	42 .6	Nucleus
FtWRKY57	FtPinGOOOO3548OO.Ol	MK161296	III	Chr.6	312	35162 .28	5.63	63.4	-0.707	55 .94	Nucleus
FtWRKY58	FtPinGOOO3O938OO.Ol	MK161341	1	Chr.7	203	22759 .07	9.65	61.82	-0.779	42 .43	Nucleus
FtWRKY59	FtPinG0002810600.01	MK161297	lld	Chr.7	130	14210.09	9.77	54.69	-0.655	73 .08	Nucleus
FtWRKYÔO	FtPinG0002864000.01	MK161328	Ma	Chr.7	322	35750.82	6.62	63.91	-0.811	44.82	Nucleus
FtWRKYÔl	FtPinG0009522600.01	MK161294	Ma	Chr.7	180	20755.01	9.13	52.44	-1.158	37.64	Nucleus
FtWRKY62	FtPinG0004132200.01	MK161317	Mb	Chr.7	508	55108.2	6.29	63.82	-0.79	46.43	Nucleus
FtWRKY63	FtPinG0004155300.01	MK161321	Ile	Chr.7	184	20475.32	5.26	32.99	-1.034	45.65	Nucleus
FtWRKY64	FtPinG0001212400.01	MK161308	III	Chr.7	305	34022.56	6.04	54.72	-0.794	57.2	Nucleus
FtWRKY65	FtPinG0007552800.01	MK161278	1	Chr.7	170	18754.78	5.97	65.29	-0.81	43.92	Nucleus
FtWRKY66	FtPinG0004764100.01	MK161330	Ile	Chr.7	292	30632.72	4.85	59.25	-0.514	53.56	Nucleus
FtWRKY67	FtPinG0002334500.01	MK161292	Mb	Chr.8	458	50521.77	5.91	54.56	-0.834	50	Nucleus
FtWRKY68	FtPinG0002325700.01	MK161303	Ile	Chr.8	273	31425.68	5.77	53.22	-1.021	58.13	Nucleus
FtWRKY69	FtPinG0002177700.01	MK161336	1	Chr.8	722	78740.04	5.59	61.02	-0.75	47.25	Nucleus
FtWRKY70	FtPinG0002142900.01	MK161302	lld	Chr.8	326	35492.1	9.69	66.99	-0.624	53.59	Nucleus
FtWRKY71	FtPinG0009255800.01	MK161314	Ile	Chr.8	247	27933.96	7.67	50.49	-0.887	63.85	Nucleus
FtWRKY72	FtPinG0005364100.01	MK161288	Mb	Chr.8	548	59507.15	5.84	64.53	-0.657	49.24	Nucleus
FtWRKY73	FtPinG0009798800.01	MK161276	Ile	Chr.8	302	32652.77	5.84	49.21	-0.766	61.06	Nucleus
FtWRKY74	FtPinG0009186100.01	MK161340	1	Chr.8	472	52360.55	6.18	51.44	-0.833	64.76	Nucleus
FtWRKY75	FtPinG0007226700.01	MK161343	Ma	Chr.8	147	16177.46	9.23	75.65	-0.396	44.21	Nucleus
FtWRKY76	FtPinG0007227700.01	MK161344	Ile	Chr.8	139	16039.78	7.67	47.63	-0.917	42.13	Nucleus
FtWRKY77	FtPinG0001770900.01	MK161324	Ile	Chr.8	270	29952.42	7.23	55.26	-0.781	56.55	Nucleus

**Figure 1:**
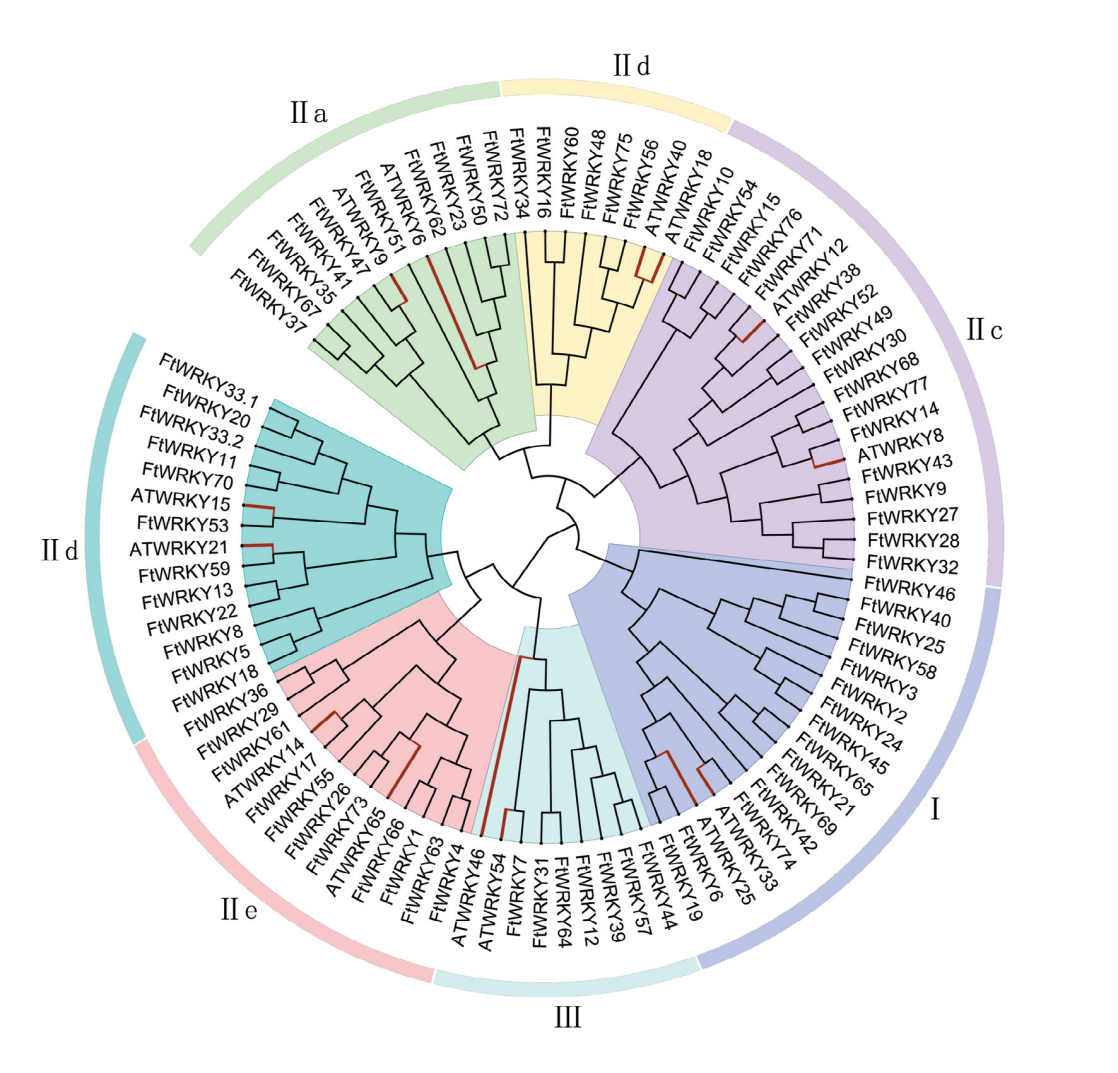
Relationship of the 78 FtWRKYs and 14 representative AtWRKYs illustrated with an unrooted phylogenetic tree. The domain sequences of all WRKYs were aligned with ClustalW and the phylogenetic tree was constructed with MEGA 6.0 software using the neighbor joining method. The branches of the AtWRKYs are in bold and highlighted *red*. The seven groups are highlighted with various colors (see color figure online).

The phylogenetic relationship of all FtWRKYs and 14 typical AtWRKYs was constructed based on multiple sequence alignment of their WRKY domains (Fig. 1) The NJ tree indicated that the FtWRKYs could be divided into three main groups defined in previous research [11]. Figure S1 showed the multiple sequence alignment of the FtWRKY domain among each group. Group II had the most FtWRKYs (56), followed by group I (15). Meanwhile, group III had a different type of zinc-finger compared with groups I and II, and contained only seven members. Group II could be further classified into five subgroups (IIa–e). Subgroup IIa (six members) and IIb (ten members) were in the same branch, while subgroup IId (12 members) and IIe (11 members) were derived from one clade. Subgroup IIc (17 members) was more similar to group I than the other subgroups. Notably, in group I, four FtWRKYs (FtWRKY3, 25, 66, and 59) had only one WRKY domain at the C-terminus, which may have lost or acquired a domain during evolution [58].

Based on the 78 complete aa sequences, we predicted the properties of the FtWRKYs, including the aa length, MW, IP, II, AI, and GRAVY (table 1). The average length of FtWRKY proteins was approximately 323.12 aa, ranging from 130 (FtWRKY59) to 722 (FtWRKY69) aa, showing a difference of approximately 4.5 times. The MW ranged from 14,210.09 (FtWRKY59) to 78,740.04 (FtWRKY69) kDa, and the IP ranged from 4.85 (FtWRKY66) to 10.04 (FtWRKY33.1). The AI varied from 32.99 (FtWRKY63) to 78.28 (FtWRKY48). Finally, the GRAVY ranged from -1.187 (FtWRKY43) to -0.396 (FtWRKY75), suggesting that all FtWRKY proteins are hydrophilic.

According to the predicted protein stability, almost 93.59% of WRKY proteins were unstable, because their II values were greater than 40, while only five FtWRKYs were stable, among which four members were from subgroup IIc. The predicted results of the subcellular localization revealed that all FtWRKYs were localized in the nucleus.

### 3.2 Gene structure and motif composition of FtWRKY proteins

**Figure 2:**
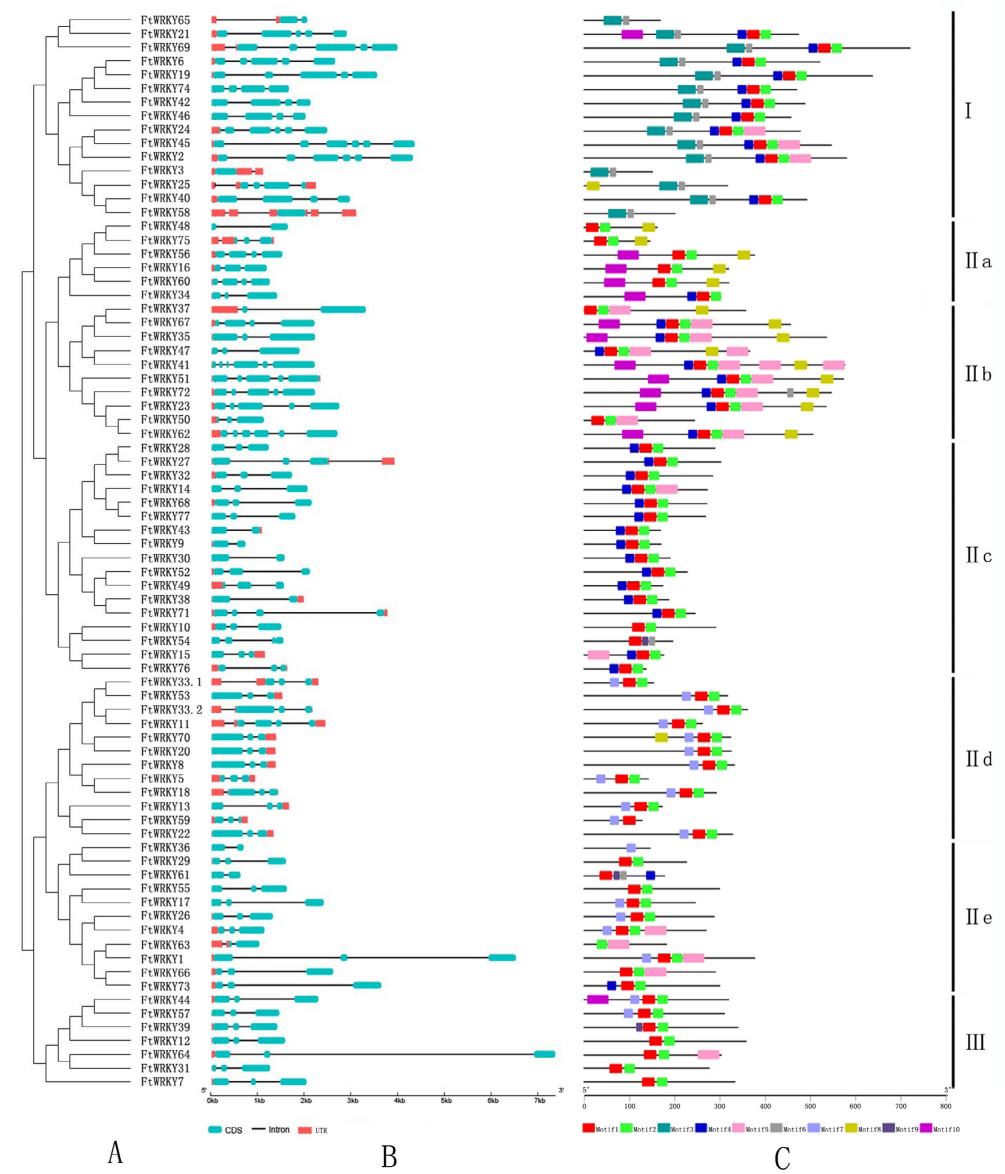
Phylogenetic relationship, gene structure, and conserved motif analysis of FtWRKYs. (A) Unrooted phylogenetic tree constructed with MEGA 6.0 software based on the 78 WRKY domain sequences with 1000 bootstrap replicates, where the colors represent different groups. (B) Gene structure of the 78 FtWRKYs predicted with GSDS, where *blue boxes* indicate exons, *red boxes* indicate untranslated regions, and *dark lines* indicate introns. The scale at the bottom can be used to estimate the lengths of the exons, introns, and untranslated regions. (C) Analysis of the conserved motif composition performed using MEME. The ten identified motifs are indicated with different colored boxes (see color figure online).

The analysis of the intron–exon structure of full-length cDNA (Fig. 2B) revealed that all FtWRKYs had introns in the translated region, although the number of introns and exons varied from one to five introns and two to six exons, respectively. The majority (52.56%) of the FtWRKY genes contained two introns. Meanwhile, six genes contained five introns and eight genes contained only one intron. Most members of subgroup IIb had two introns, except FtWRKY23 and 47, and five members of group III (71.43%) contained three exons and two introns. Genes with similar structures were always clustered in the same group, which was further confirmed by the results of the FtWRKY classification (Fig. 2A).

Ten motifs were identified in Tartary buckwheat using MEME software. Table S4 presents detailed information of these motifs and Figure 2C presents the motif compositions of each FtWRKY. The length of the 10 motifs ranged from 15 (motif 9) to 49 (motif 5) bp. Motifs 3 and 6 partly represented the distribution of the conserved domain at the C-terminus and were shared by all 15 members of group I. Meanwhile, motifs 1 and 2 partly represented the distribution of the conserved domain at the N-terminus and were found in the majority of FtWRKYs. The other six motifs appeared around the WRKY domain, but were distributed uniquely, and their function remains unclear. In group I, 11 of 15 FtWRKYs contained motifs 1, 2, and 4, while FtWRKY3, 25, 58, and 65 contained only one WRKY domain at the C-terminus. These three motifs were also found in 15 of the 17 proteins of subgroup IIc, because FtWRKY54 lacked the zinc-finger-like motif and FtWRKY10 did not contain motif 4. Similarly, in subgroup IId, motifs 1, 2, and 7 were found in 11 of the 12 members, except the one member without a zinc finger (FtWRKY59). This indicated that the motifs were selectively distributed among the groups. Motif 8 was found in all members of subgroup IIa and nine of the ten members of subgroup IIb. Moreover, all members in subgroup IIb contained motif 5. Motif 10 was mostly found in subgroups IIa and IIb, while motif 9 was only identified in FtWRKY39, 54, and 61. Overall, the variety and complexity of these motifs suggests that these proteins likely have additional functions [59].

### 3.3 Chromosomal locations of FtWRKY genes

**Figure 3:**
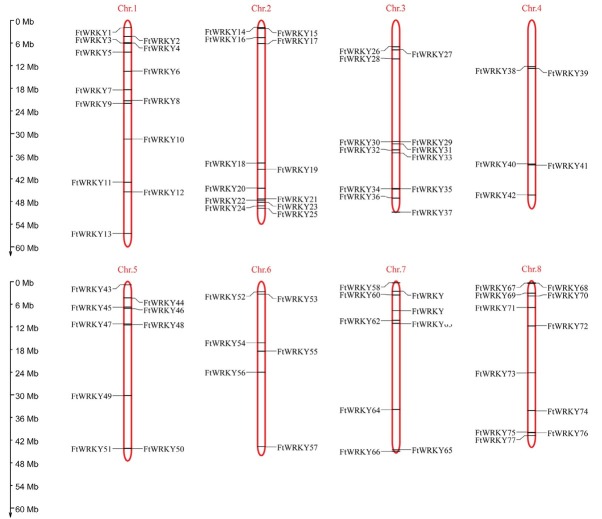
Location and distribution of the FtWRKY genes in all eight chromosomes mapped based on their genomic position.

All 77 FtWRKY genes were separately mapped onto the eight chromosomes of Tartary buckwheat (Chr.1–8). Most FtWRKY genes were observed at the top and bottom arms of the chromosomes (Fig. 3). Chr.1 contained the most FtWRKY genes, with 13 out of 77 genes (16.88%), whereas Chr.4 contained the fewest, with five genes (6.49%). Chr.2 and Chr.3 each contained 12 genes (15.58%), while Chr.5 and Chr.7 each contained nine genes (11.69%). In addition, Chr.6 and Chr.8 contained six (7.79%) and 11 genes (14.29%), respectively. Each chromosome contained more than five classes of FtWRKYs. The FtWRKYs of subgroup IIc were the most widely distributed, and found on seven chromosomes (except Chr.7), while each of the other groups were located on six chromosomes. Interestingly, the FtWRKYs of subgroups IId and IIe were located on the same chromosomes, except Chr.4 and Chr.5. The results revealed a non-uniform distribution of WRKY genes among the chromosomes of Tartary buckwheat.

### 3.4 Duplication and evolution of FtWRKY

Gene duplication plays a vital role in the enlargement of gene families [60]. Chromosome regions shorter than 100 kb containing more than two genes with a similarity greater than 40% are considered to be caused by a tandem duplication event [61]. We found two pairs of tandem duplicates, FtWRKY29 and 30 and FtWRKY50 and 51,

which were located on Chr.3 and Chr.5, respectively. Both pairs of tandem duplicates were found in group II.

We calculated the Ka/Ks ratios of the orthologous pairs between Tartary buckwheat and common buckwheat (Table 2) and found 63 orthologous gene-pairs of WRKY TFs. The Ka/Ks values ranged from 0.0626 to 1.1014 (average: 0.2833). The overwhelming majority of gene pairs (62 pairs) had Ka/Ks ratios < 1, indicative of strong purifying selection acting on these genes, while only one pair had a Ka/Ks ratio > 1 (FtWRKY34 and Fes_ sc0001280.1.g000011.aua.1). Moreover, none of the genes had Ka/Ks ratios of ~1, indicating that neutral selection did not occur. The divergence time of Tartary and common buckwheat was predicted to have occurred between 1.630 and 17.623 Mya.

**Table 2:** Ka/Ks ratios and estimated divergence times of orthologous WRKY proteins in Tartary buckwheat and common buckwheat.

Protein names (Taraty buckwheat)	Protein IDs (buckwheat)	Ks	Ka	Ka/Ks	Time^a^ (MYA)
FtWRKY2	Fes_sc0008092.1.g000001.aua.1	0.0933	0.0123	0.1322	3.110
FtWRKY57	Fes_sc0004855.1.g000007.aua.1	0.35	0.1955	0.5587	11.667
FtWRKY43	Fes_sc0004942.1.g000002.aua.1	0.4657	0.051	0.1096	15.523
FtWRKY64	Fes_sc0000377.1.g000010.aua.1	0.501	0.1124	0.2243	16.700
FtWRKY3	Fes_sc0011045.1.g000001.aua.1	0.5287	0.235	0.4444	17.623
FtWRKY47	Fes_sc0001199.1.g000019.aua.1	0.3288	0.1271	0.3866	10.960
FtWRKY6	Fes_sc0000044.1.g000018.aua.1	0.1673	0.0252	0.1505	5.577
FtWRKY33.2	Fes_sc0000926.1.g000002.aua.1	0.087	0.0158	0.1814	2.900
FtWRKY77	Fes_sc0013031.1.g000003.aua.1	0.2838	0.0438	0.1542	9.460
FtWRKY8	Fes_sc0014831.1.g000001.aua.1	0.1268	0.0355	0.2803	4.227
FtWRKY70	Fes_sc0000037.1.g000002.aua.1	0.2145	0.0301	0.1404	7.150
FtWRKY69	Fes_sc0000011.1.g000128.aua.1	0.0818	0.0284	0.347	2.727
FtWRKY67	Fes_sc0009561.1.g000007.aua.1	0.17	0.058	0.3411	5.667
FtWRKY4	Fes_sc0008506.1.g000001.aua.1	0.1878	0.0351	0.1872	6.260
FtWRKY44	Fes_sc0005050.1.g000005.aua.1	0.1876	0.0523	0.2788	6.253
FtWRKY52	Fes_sc0006770.1.g000001.aua.1	0.5152	0.1731	0.336	17.173
FtWRKY53	Fes_sc0069080.1.g000001.aua.1	0.1253	0.0159	0.1268	4.177
FtWRKY60	Fes_sc0000003.1.g000030.aua.1	0.2816	0.1011	0.3588	9.387
FtWRKY20	Fes_sc0029985.1.g000001.aua.1	0.1534	0.0253	0.1646	5.113
FtWRKY12	Fes_sc0011190.1.g000002.aua.1	0.2406	0.1457	0.6054	8.020
FtWRKY5	Fes_sc0003720.1.g000003.aua.1	0.2283	0.0331	0.1449	7.610
FtWRKY21	Fes_sc0007586.1.g000004.aua.1	0.1271	0.05	0.3939	4.237
FtWRKY22	Fes_sc0011986.1.g000003.aua.1	0.1273	0.0175	0.1378	4.243
FtWRKY25	Fes_sc0000035.1.g000037.aua.1	0.1216	0.0589	0.484	4.053
FtWRKY62	Fes_sc0000187.1.g000019.aua.1	0.1237	0.0178	0.1438	4.123
FtWRKY63	Fes_sc0000026.1.g000039.aua.1	0.0489	0.0072	0.1473	1.630
FtWRKY48	Fes_sc0000472.1.g000012.aua.1	0.2265	0.0915	0.4037	7.550
FtWRKY13	Fes_sc0029335.1.g000001.aua.1	0.2211	0.0304	0.1376	7.370
FtWRKY35	Fes_sc0008226.1.g000001.aua.1	0.194	0.049	0.2528	6.467
FtWRKY34	Fes_sc0001280.1.g000011.aua.1	0.1692	0.1863	1.1014	5.640
FtWRKY45	Fes_sc0001079.1.g000012.aua.1	0.1131	0.0374	0.3307	3.770
FtWRKY46	Fes_sc0002930.1.g000006.aua.1	0.1755	0.0355	0.2023	5.850
FtWRKY66	Fes_sc0005571.1.g000002.aua.1	0.0956	0.0258	0.2703	3.187
FtWRKY26	Fes_sc0012248.1.g000002.aua.1	0.2519	0.089	0.3535	8.397
FtWRKY7	Fes_sc0015209.1.g000003.aua.1	0.1533	0.0635	0.4143	5.110
FtWRKY19	Fes_sc0004112.1.g000005.aua.1	0.1388	0.0408	0.2941	4.627
FtWRKY15	Fes_sc0019573.1.g000002.aua.1	0.2846	0.0344	0.1209	9.487
FtWRKY30	Fes_sc0002003.1.g000003.aua.1	0.3094	0.1056	0.3413	10.313
FtWRKY29	Fes_sc0039378.1.g000001.aua.1	0.4246	0.1549	0.3649	14.153
FtWRKY41	Fes_sc0000009.1.g000030.aua.1	0.1215	0.0138	0.114	4.050
FtWRKY40	Fes_sc0000009.1.g000064.aua.1	0.1256	0.0211	0.1676	4.187
FtWRKY72	Fes_sc0002754.1.g000009.aua.1	0.3706	0.0839	0.2265	12.353
FtWRKY9	Fes_sc0004559.1.g000004.aua.1	0.2475	0.0377	0.1523	8.250
FtWRKY16	Fes_sc0001592.1.g000015.aua.1	0.1999	0.0477	0.2386	6.663
FtWRKY10	Fes_sc0007337.1.g000004.aua.1	0.1715	0.0807	0.4707	5.717
FtWRKY27	Fes_sc0003240.1.g000006.aua.1	0.1237	0.0468	0.3786	4.123
FtWRKY51	Fes_sc0045576.1.g000001.aua.1	0.2371	0.0421	0.1775	7.903
FtWRKY50	Fes_sc0014867.1.g000001.aua.1	0.155	0.1329	0.8579	5.167
FtWRKY42	Fes_sc0000437.1.g000022.aua.1	0.1495	0.0318	0.2124	4.983
FtWRKY24	Fes_sc0001536.1.g000007.aua.1	0.1069	0.028	0.2618	3.563
FtWRKY17	Fes_sc0003509.1.g000001.aua.1	0.2287	0.0523	0.2285	7.623
FtWRKY75	Fes_sc0008827.1.g000003.aua.1	0.2043	0.0441	0.2159	6.810
FtWRKY18	Fes_sc0001529.1.g000004.aua.1	0.4757	0.1315	0.2764	15.857
FtWRKY28	Fes_sc0038083.1.g000001.aua.1	0.3283	0.0723	0.2201	10.943
FtWRKY65	Fes_sc0001852.1.g000019.aua.1	0.1255	0.0274	0.2182	4.183
FtWRKY39	Fes_sc0001496.1.g000021.aua.1	0.1397	0.0392	0.2805	4.657
FtWRKY49	Fes_sc0008656.1.g000006.aua.1	0.2406	0.0151	0.0626	8.020
FtWRKY38	Fes_sc0000408.1.g000020.aua.1	0.2339	0.0355	0.1517	7.797
FtWRKY56	Fes_sc0000224.1.g000018.aua.1	0.2222	0.0278	0.125	7.407
FtWRKY14	Fes_sc0012847.1.g000001.aua.1	0.2519	0.0525	0.2083	8.397
FtWRKY74	Fes_sc0004443.1.g000014.aua.1	0.2923	0.0539	0.1845	9.743
FtWRKY61	Fes_sc0000022.1.g000019.aua.1	0.1677	0.1266	0.7551	5.590
FtWRKY73	Fes_sc0000894.1.g000012.aua.1	0.0982	0.0115	0.1166	3.273

### 3.5 Expression patterns of FtWRKY in different tissue and under salt treatment

**Figure 4:**
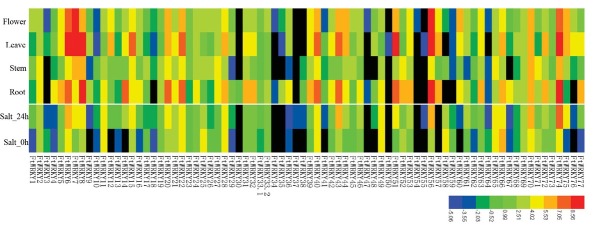
Expression profile of FtWRKY genes among different tissues (i.e. root, stem, leaf and flower) and under salt stress. Levels of transcript accumulation from low to high are shown by using the color from blue to red. (see color figure online).

The heatmap of 78 FtWRKYs was constructed by log2 transformed FPKM values (Fig.4). FtWRKY37, 50, 54 and 55 were barely expressed in any of the selected tissues. Other 74 of 78 (94.9%) FtWRKYs were expressed at least in one tissue. Some genes, like FtWRKY8, 70 and 74, were highly expressed in all the tissues, suggesting these FtWRKYs may play an important role in the plant development of Tartary buckwheat. Additionally, some genes showed high expression simultaneously in flower, leaf and root, such as FtWRKY6, 43 and 51, while most of the genes showed lower expression levels in stem. Interestingly, a small number of genes presented tissue-specific expression profiling, for example, FtWRKY14 was only particularly expressed in root, and it may play key role in this tissue.

A total of 71 FtWRKYs (91.03%) were detected as being expressed under salt stress, indicating that most FtWRKYs genes were related to dealing with environment change. In total, 50 genes were upregulated, among these FtWRKY74 and 6 were most significantly followed by FtWRKY43 and 56.

### 3.6 Expression analysis of selected FtWRKYs under abiotic stress

**Figure 5:**
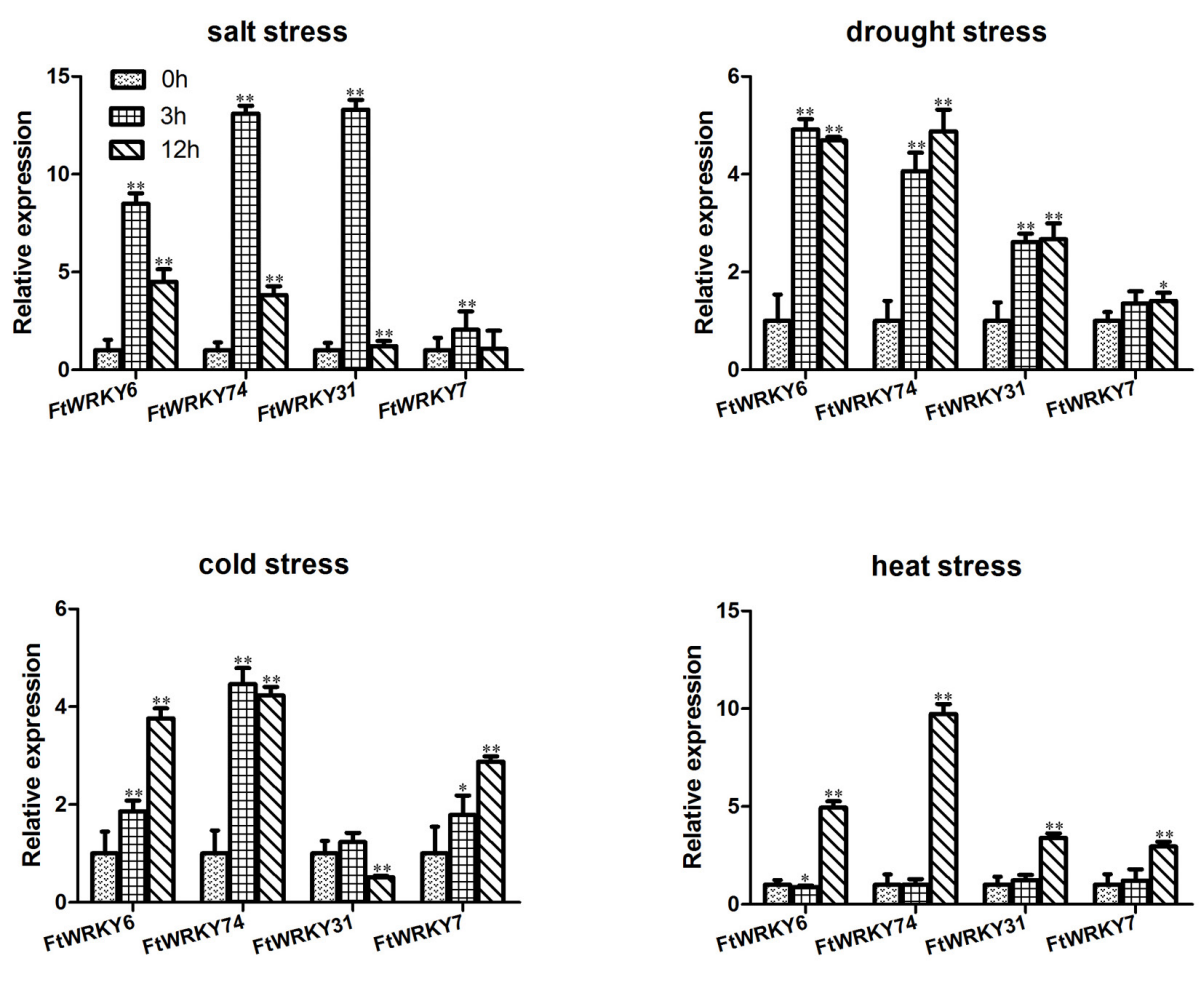
Relative expression patterns of four selected FtWRKY genes under four abiotic stresses. Results are presented as the means ± standard deviation. Samples were collected at 0, 3, and 12 h, and 0 h was used as the control. *Asterisks* indicate the gene significantly upregulated or downregulated under abiotic stresses using *t* text(* *p*<0.05, **p<0.01). (A) Relative expression under drought treatment (30% PEG6000). (B) Relative expression under salinity treatment (15% NaCl). (C) Relative expression under cold treatment (4°C, growth chamber). (D) Relative expression under heat treatment (40°C, growth chamber).

TFs typically contain various types of DNA-binding domains and can improve expression at the transcription level under different abiotic stresses. Because the members of the same subgroups exhibit functional similarities [13], we selected four FtWRKYs (6, 7, 31, and 74) for an abiotic stress expression study in seedlings (Fig. 5), which were clustered in same groups with AtWRKY25, 33, 46, and 54.

Under salt stress, all FtWRKY genes were upregulated during the first 3 h and then decreased, while FtWRKY6, 74, and 31 were significantly up-regulated. The highest

expression (13-fold increase) was detected after 3 h in FtWRKY31, followed by FtWRKY74. Three FtWRKY genes (FtWRKY74, 31, and 7) were significantly induced by drought conditions, and maintained a highly increased expression of at least 2.67-fold until 24 h. Meanwhile, FtWRKY6 showed an almost 5-fold increase within 3 h, but decreased a few hours later. Under cold treatment, the relative expression of FtWRKY74 increased by more than 4.5-fold within 3 h, while the expression of FtWRKY6 and 7 was significantly upregulated at the 12 h. Meanwhile, FtWRKY31 fluctuated slightly with an upregulation of less than 1.3-fold after 3 h and downregulation below that of the control after 12 h. All genes exhibited similar expression patterns under heat treatment, with no clear changes within 3 h and significantly upregulation of at least 2.95-fold within 12 h. FtWRKY74 showed the highest expression (almost 10-fold increase), whereas FtWRKY7 showed only a small change.

All selected FtWRKY genes responded to the abiotic stress treatments. FtWRKY6 and 74 showed rapid and significant upregulation with all treatments within 3 h. Under heat treatment, all genes were induced after 3 h. Except for FtWRKY31 under cold treatment within 12 h and FtWRKY6 under heat treatment within 3 h, the expression levels of all genes under the various stresses showed greater fold-changes than the control. Within 3 h, the average fold-change with salt treatment was 9.24-fold, which was the most significant change, but only 1.09-fold with heat treatment. Meanwhile, the average fold-changes were 3.24 and 2.34 under drought and cold conditions, respectively. By contrast, after 12 h, the heat condition showed the highest average fold change (5.26-fold), while the salt treatment induced a fold-change of only 2.66. In addition, the drought and cold treatments caused fold-changes of 3.41 and 2.85 within 12 h, respectively.

## 4 Discussion

The WRKY family is one of the largest TF families in higher plants, and its members play an essential role in many physiological processes. In this study, a total of 78 FtWRKYs were identified, and all proteins presented clear differences, suggesting a high degree of complexity among FtWRKYs. The 78 FtWRKYs could be divided into three main groups, which was consistent with previous studies [6, 8]. Comparing with other species (Table S1), the number in Tartary buckwheat (78 FtWRKYs) was closest to that of *Arabidopsis* (72 AtWRKYs) [17]. Therefore, they likely underwent similar evolutionary patterns. Interestingly, Group II was always the largest group among the species (e.g., 70.5% in Tartary buckwheat and 69.1% in soybean), suggesting that group II may have undergone significant expansion during the course of evolution and that group II mainly accounts for the diversity of the WRKY family among species.

Despite the high conservation of the WRKYGQK sequence in the WRKY family, there were still some interesting cases. The WRKYGKK sequence present in FtWRKY15 and 76 was similar to those identified in many species, such as tomato, apple, and peach [62, 63, 64], this kind may have lost their ability to combine with the W-box [28]. Meanwhile, the variant type of WRKYDQK was only observed in FtWRKY50, and has also been reported in carrot [65]. Similar to other plants, the properties of FtWRKY proteins showed differences among groups and individuals [11].

From the phylogenetic tree, we identified at least one FtWRKY and AtWRKY in each subgroup, illustrating that the differentiation time of the WRKY family was earlier than the divergence time of Tartary buckwheat and *A. thaliana*. The FtWRKY members of the specific subgroups likely shared closely related motif compositions and functional similarities, which was supported by the subsequent gene structure analysis. Meanwhile, genes containing six exons were clustered in groups I and II, indicating that groups I and II constituted the ancestral genes.

Gene duplication plays a vital role in the enlargement of gene families [60]. Although a whole-genome duplication event occurred in Tartary buckwheat [42], only two pairs of tandem duplicates were found in the FtWRKY family, FtWRKY29 and 30 (90% similarity) and FtWRKY 50 and 51 (44% similarity). Both pairs of tandem duplicates were found in group II. By contrast, most duplication events in *Arabidopsis* and rice are found in group III. Surprisingly, no segmental duplications were identified in the FtWRKYs, therefore, duplication likely had a limited contribution to the expansion of the WRKY family in Tartary buckwheat. At the same time, a total of 63 orthologous gene-pairs between Tartary buckwheat and common buckwheat were identified, and the Ka/Ks ratios of 62 to 63 pairs (98.41%) were < 1, indicative of strong purifying selection acting on these genes.

Like the expression pattern of WRKY in other species, different genes showed incongruous expression patterns, suggesting they have various function and

diversity regulatory mechanisms [12, 66]. According to the RNA-seq data of Tartary buckwheat, some genes were not expressed in any tissues, the reason may be some of them are pseudogenes, or they were expressed in other tissues we failed to collect. FtWRKY74 and 6 showed the highest expression level after salt treatment, which revealed these genes can improve the tolerance under osmotic stress, therefore were selected as the candidate genes to develop qRT-PCR analysis.

Substantial evidence has shown that WRKY TFs can improve the stress tolerance of plants by modulating their molecular and physiological metabolism [8, 20]. In *Arabidopsis*, at least 26 AtWRKY genes have been demonstrated to participate in abiotic stress responses [67]. For example, overexpression of AtWRKY25 and 33 was reported under heat and salt treatment [32]. The expression pattern results suggested that the four assessed FtWRKYs were involved in the regulation of abiotic stress responses. Under heat treatment, all four genes were upregulated within 12 h, indicating that the WRKY TFs participated in a complex cross-regulation network under such stresses. FtWRKY6 and 74 simultaneously responded to salt and drought treatments, suggesting that they can co-regulate more than one adverse condition, possibly based on synergistic or antagonistic mechanisms. Overall, the results indicate that the selected four FtWRKY genes play an important role in the establishment of salt, drought, cold, and heat tolerance.

Furthermore, it is indicative of the selection method of responsive genes in our study is efficient. RNA-sequencing analysis can help us to find the genes with higher expression under stress, while sequence alignment suggest the genes clustered with the reported stress resistance genes have similar functions.

## 5 Conclusions

We identified 78 FtWRKY proteins (77 genes) from the *F*. *tataricum* genome sequence, which could be divided into three groups. Although the proteins were diverse, the members in same groups and subgroups exhibited similar properties, such as gene structure and motif composition. The results suggested that duplication events contributed little to the expansion and evolution of FtWRKYs, and most FtWRKYs experienced purifying selection during the course of evolution. Finally, the expression analysis indicated that all four studied FtWRKYs (6, 74, 31, and 7) were regulated by abiotic stresses. In particular, FtWRKY6 and 74 were highly responsive to both salt and drought treatment. Based on results above, these four selected genes show potential for transgenic applications in Tartary buckwheat. This study is the solid foundation for expression analyses of additional FtWRKYs and related mechanistic studies, and can prove the fundamental theory to clone specific functional genes.

## References

[1] Lawlor D (2011). Abiotic Stress Adaptation in Plants. Physiological, Molecular and Genomic Foundation. Ann Bot.

[2] Chinnusamy V, Schumaker K, Zhu JK (2004). Molecular genetic perspectives on cross-talk and specificity in abiotic stress signalling in plants. J Exp Bot.

[3] Xu Y, Liu F, Han G, Cheng B (2018). Genome-wide identification and comparative analysis of phosphate starvation-responsive transcription factors in maize and three other gramineous plants. Plant Cell Rep.

[4] Pan Y, Tsai CJ, Ma B (2010). Nussinov, R. Mechanisms of transcription factor selectivity. Trends Genet.

[5] Baloglu MC, Eldem V, Hajyzadeh M, Unver T (2014). Genome-Wide Analysis of the bZIP Transcription Factors in Cucumber. PloS One.

[6] Liu QN, Liu Y, Xin ZZ, Zhang DZ, Ge BM, Yang RP (2017). Genome-wide identification and characterization of the WRKY gene family in potato Solanum tuberosum. Biochem Syst Ecol.

[7] Zhang Y, Yu HJ, Yang XY, Li Q, Ling J, Wang H (2016). CsWRKY46 a WRKY transcription factor from cucumber, confers cold resistance in transgenic-plant by regulating a set of cold-stress responsive genes in an ABA-dependent manner. Plant Physiol Biochem.

[8] Karanja BK, Fan L, Liang X, Yan W, Zhu X, Tang M (2017). Genome-wide characterization of the WRKY gene family in radish ( Raphanus sativus L.) reveals its critical functions under different abiotic stresses. Plant Cell Rep.

[9] Zhao H, Wang S, Chen S, Jiang J, Liu G (2015). Phylogenetic and stress-responsive expression analysis of 20 WRKY genes in Populus simonii×Populus nigra. Gene.

[10] He HS, Dong Q, Shao YH, Jiang HY, Zhu SW, Cheng BJ (2012). Genome-wide survey and characterization of the WRKY gene family in Populus trichocarpa. Plant Cell Rep.

[11] Eulgem T, Rushton PJ, Robatzek S, Somssich IE (2000). The WRKY superfamily of plant transcription factors. Trends in Plant Sci.

[12] Jiang Y, Duan Y, Yin J, Ye S, Zhu J, Zhang F (2014). Genome-wide identification and characterization of the Populus WRKY transcription factor family and analysis of their expression in response to biotic and abiotic stresses. J Exp Bot.

[13] Rushton PJ, Somssich IE, Ringler P, Shen QJ (2010). WRKY transcription factors. Trends Plant Sci.

[14] Brand LH, Kirchler T, Hummel S, Chaban C, Wanke D (2010). DPI-ELISA: a fast and versatile method to specify the binding of plant transcription factors to DNA in vitro. Plant Methods.

[15] Wang N, Xia EH, Gao LZ (2016). Genome-wide analysis of WRKY family of transcription factors in common bean, Phaseolus vulgaris : Chromosomal localization, structure, evolution and expression divergence. Plant Gene.

[16] Ishiguro S, Nakamura K (1994). Characterization of a cDNA encoding a novel DNA-binding protein, SPF1, that recognizes SP8 sequences in the 5’ upstream regions of genes coding for sporamin and beta-amylase from sweet potato. Mol Gen Genet.

[17] Wu KL, Guo ZJ, Wang HH, Li J (2005). The WRKY Family of Transcription Factors in Rice and Arabidopsis and Their Origins. DNA Res.

[18] Zhu X, Liu S, Chen M, Qin L, Kong L, Xia G (2013). WRKY transcription factors in wheat and their induction by biotic and abiotic stress. Plant Mol Biol Rep.

[19] Wu ZJ, Li XH, Liu ZW, Li H, Wang YX, Zhuang J (2016). Transcriptome-wide identification of Camellia sinensis WRKY transcription factors in response to temperature stress. Mol Genet Genomics.

[20] Yu Y, Nan W, Hu R, Xiang F (2016). Genome-wide identification of soybean WRKY transcription factors in response to salt stress. Springerplus.

[21] Jiang W, Yu D (2009). Arabidopsis WRKY2 transcription factor mediates seed germination and postgermination arrest of development by abscisic acid. BMC Plant Biol.

[22] Ay N, Irmler K, Fischer A, Uhlemann R, Reuter G, Humbeck K (2009). Epigenetic programming via histone methylation at WRKY53 controls leaf senescence in Arabidopsis thaliana. Plant J.

[23] Liu D, Leib K, Zhao P, Kogel KH, Langen G (2014). Phylogenetic analysis of barley WRKY proteins and characterization of HvWRKY1 and -2 as repressors of the pathogen-inducible gene HvGER4c. Mol Genet Genomics.

[24] Atamian HS, Eulgem T, Kaloshian I (2012). SlWRKY70 is required for Mi-1-mediated resistance to aphids and nematodes in tomato. Planta.

[25] Okay S, Derelli E, Unver T (2014). Transcriptome-wide identification of bread wheat WRKY transcription factors in response to drought stress. Mol Genet Genomics.

[26] Dang FF, Wang YN, Yu L, Eulgem T, Lai Y, Liu ZQ (2013). CaWRKY40, a WRKY protein of pepper, plays an important role in the regulation of tolerance to heat stress and resistance to Ralstonia solanacearum infection. Plant Cell Environ.

[27] Kayum MA, Jung HJ, Park JI, Ahmed NU, Saha G, Yang TJ (2015). Identification and expression analysis of WRKY family genes under biotic and abiotic stresses in Brassica rapa. Mol Genet Genomics.

[28] Xiong W, Xu X, Zhang L, Wu P, Chen Y, Li M (2013). Genome-wide analysis of the WRKY gene family in physic nut Jatropha curcas L.). Gene.

[29] Wang F, Hou X, Tang J, Wang Z, Wang S, Jiang F (2012). A novel cold-inducible gene from Pak-choi Brassica campestris ssp chinensis BcWRKY46, enhances the cold, salt and dehydration stress tolerance in transgenic tobacco. Mol Biol Rep.

[30] Yang G, Chao W, Wang Y, Guo Y, Zhao Y, Yang C (2016). Overex-pression of ThVHAc1 and its potential upstream regulator, ThWRKY7 improved plant tolerance of Cadmium stress. Sci Rep.

[31] Li J, Besseau S, Törönen P, Sipari N, Kollist H, Holm L (2013). Defense-related transcription factors WRKY70 and WRKY54 modulate osmotic stress tolerance by regulating stomatal aperture in Arabidopsis. New Phytol.

[32] Jiang Y, Deyholos M (2009). Functional characterization of Arabidopsis NaCl-inducible WRKY25 and WRKY33 TFs in abiotic stresses. Plant Mol Biol.

[33] Li S, Zhang P, Zhang M, Fu C, Yu L (2013). Functional analysis of a WRKY transcription factor involved in transcriptional activation of the DBAT gene in Taxus chinensis. Plant Biol.

[34] Mishra S, Triptahi V, Singh S, Phukan UJ, Gupta MM, Shanker K (2013). Wound induced tanscriptional regulation of benzylisoquinoline pathway and characterization of wound inducible PsWRKY transcription factor from Papaver somniferum. PloS One.

[35] Zhu F (2016). Chemical composition and health effects of Tartary buckwheat. Food Chem.

[36] Ren Q, Wu C, Ren Y, Zhang J (2013). Characterization and identification of the chemical constituents from tartary buckwheat ( Fagopyrum tataricum Gaertn) by high performance liquid chromatography/photodiode array detector/linear ion trap FTICR hybrid mass spectrometry. Food Chem.

[37] Zhao LJ, Zhang ZW, Yu LI, Wang TY (2006). Genetic Diversity in Tartary Buckwheat Based on ISSR Markers. J Plant Genet Resources.

[38] Zhou M, Wang C, Qi L, Yang X, Sun Z, Tang Y (2015). Ectopic Expression of Fagopyrum tataricum FtMYB12 Improves Cold Tolerance in Arabidopsis thaliana. J Plant Growth Regul.

[39] Cai Y, Luo Q, Sun M, Corke H (2004). Antioxidant activity and phenolic compounds of 112 traditional Chinese medicinal plants associated with anticancer. Life Sci.

[40] Gao F, Zhao HX, Yao HP, Li CL, Chen H, Wang AH (2016). Identification, isolation and expression analysis of eight stress-related R2R3-MYB genes in tartary buckwheat Fagopyrum tataricum. Plant Cell Rep.

[41] Fabjan N, Rode J, Kosir IJ, Wang Z, Zhang Z, Kreft I (2003). Tartary buckwheat Fagopyrum tataricum Gaertn.) as a source of dietary rutin and quercitrin. J Agric Food Chem.

[42] Zhang L, Li X, Ma B, Gao Q, Du H, Han Y (2017). The Tartary Buckwheat Genome Provides Insights into Rutin Biosynthesis and Abiotic Stress Tolerance. Mol Plant.

[43] Karki R, Park CH, Kim DW (2013). Extract of buckwheat sprouts scavenges oxidation and inhibits pro-inflammatory mediators in lipopolysaccharide-stimulated macrophages (RAW264.7). J Integr Med.

[44] Gaberscik A, Voncina M, Trost T, Germ M, Bjorn LO (2002). Growth and production of buckwheat Fagopyrum esculentum treated with reduced, ambient, and enhanced UV-B radiation. J Photochem Photobiol, B.

[45] Finn RD, John T, Jaina M, Coggill PC, John SS, Hans-Rudolf H (2008). The Pfam protein families database. Nucleic Acids Res.

[46] Letunic I, Doerks T, Bork P (2012). SMART 7: recent updates to the protein domain annotation resource. Nucleic Acids Res.

[47] Rhee SY, Beavis W., Berardini T. Z., Chen G., Dixon D., Doyle A. (2003). The Arabidopsis information resource (TAIR) : a model organism database providing a centralized, curated gateway to Arabidopsis biology, research materials and community. Nucleic Acids Res.

[48] Thompson JD, Gibson TJ, Plewniak F, Jeanmougin F, Higgins DG (1997). The CLUSTAL_X windows interface: flexible strategies for multiple sequence alignment aided by quality analysis tools. Nucleic Acids Res.

[49] Tamura K, Stecher G, Peterson D, Filipski A, Kumar S (2013). MEGA6: Molecular Evolutionary Genetics Analysis Version 6.0. Mol Biol Evol.

[50] Chou KC, Shen HB (2010). Cell-PLoc 2.0: an improved package of web-servers for predicting subcellular localization of proteins in various organisms. Nat Sci.

[51] Hu B, Jin J, Guo A Y, Zhang H, Luo J, Gao G (2015). GSDS 2.0: an upgraded gene feature visualization server. Bioinformatics.

[52] Liu W, Zhang Z, Li W, Zhu W, Ren Z, Wang Z (2018). Genome-wide identification and comparative analysis of the 3-hydroxy-3-methylglutaryl coenzyme a reductase (HMGR) gene family in Gossypium. Molecules.

[53] Blanc G, Wolfe KH (2004). Widespread paleopolyploidy in model plant species inferred from age distributions of duplicate genes. Plant Cell.

[54] Zhao P, Wang D, Wang R, Kong N, Zhang C, Yang C (2018). Genome-wide analysis of the potato hsp20 gene family: identification, genomic organization and expression profiles in response to heat stress. Bmc Genomics.

[55] Bai YC, Li CL, Zhang JW, Li SJ, Luo XP, Yao HP (2014). Characterization of two tartary buckwheat R2R3-MYB transcription factors and their regulation of proanthocyanidin biosynthesis. Physiol Plant.

[56] Chandra S, Kazmi AZ, Ahmed Z, Roychowdhury G, Kumari V, Kumar M (2017). Genome-wide identification and characterization of NB-ARC resistant genes in wheat ( Triticum aestivum L.) and their expression during leaf rust infection. Plant Cell Rep.

[57] Diao WP, Snyder JC, Wang SB, Liu JB, Pan BG, Guo GJ (2016). Genome-Wide Identification and Expression Analysis of WRKY Gene Family in Capsicum annuum L. Front Plant Sci.

[58] Ross CA, Liu Y, Shen QJ (2007). The WRKY Gene Family in Rice Oryza sativa. J Integr Plant Biol.

[59] Puranik S, Sahu PP, Srivastava PS, Prasad M (2012). NAC proteins: regulation and role in stress tolerance. Trends Plant Sci.

[60] Vision TJ, Brown DG, Tanksley SD (2000). The origins of genomic duplications in Arabidopsis. Science.

[61] Kong WL, Yu K, Dan NZ, Yang SZ, Bao MZ, Fu XP (2017). Genome-wide identification and expression analysis of WRKY transcription factor under abiotic stress in Beta vulgaris. Sci Agric Sin.

[62] Huang S, Gao Y, Liu J, Peng X, Niu X, Fei Z (2012). Genome-wide analysis of WRKY transcription factors in Solanum lycopersicum. Mol Genet Genomics.

[63] Meng D, Li Y, Bai Y, Li M, Cheng L (2016). Genome-wide identification and characterization of WRKY transcriptional factor family in apple and analysis of their responses to waterlogging and drought stress. Plant Physiol Biochem.

[64] Chen M, Tan Q, Sun M, Li D, Fu X, Chen X (2016). Genome-wide identification of WRKY family genes in peach and analysis of WRKY expression during bud dormancy. Mol Genet Genomics.

[65] Li MY, Xu ZS, Tian C, Huang Y, Wang F, Xiong AS (2016). Genomic identification of WRKY transcription factors in carrot Daucus carota and analysis of evolution and homologous groups for plants. Sci Rep.

[66] Ma J, Lu J, Xu J, Duan B, He X, Liu J (2015). Genome-wide identification of wrky genes in the desert poplar Populus euphratica and adaptive evolution of the genes in response to salt stress. Evol Bioinf.

[67] Jiang Y, Deyholos MK (2006). Comprehensive transcriptional profiling of NaCl-stressed Arabidopsis roots reveals novel classes of responsive genes. BMC Plant Biol.

