# Supplementary material for: Genome-wide Analysis of the WRKY Gene Family and its Response to Abiotic Stress in Buckwheat (Fagopyrum Tataricum)
Source: Open Life Sci. 2019 Mar 20;14:80–96. doi: 10.1515/biol-2019-0010 (PMC7874777; doi:10.1515/biol-2019-0010)
Supplement: Supplementary file 1 [file biol-14-080_sm.pdf]

Electronic supplementary material

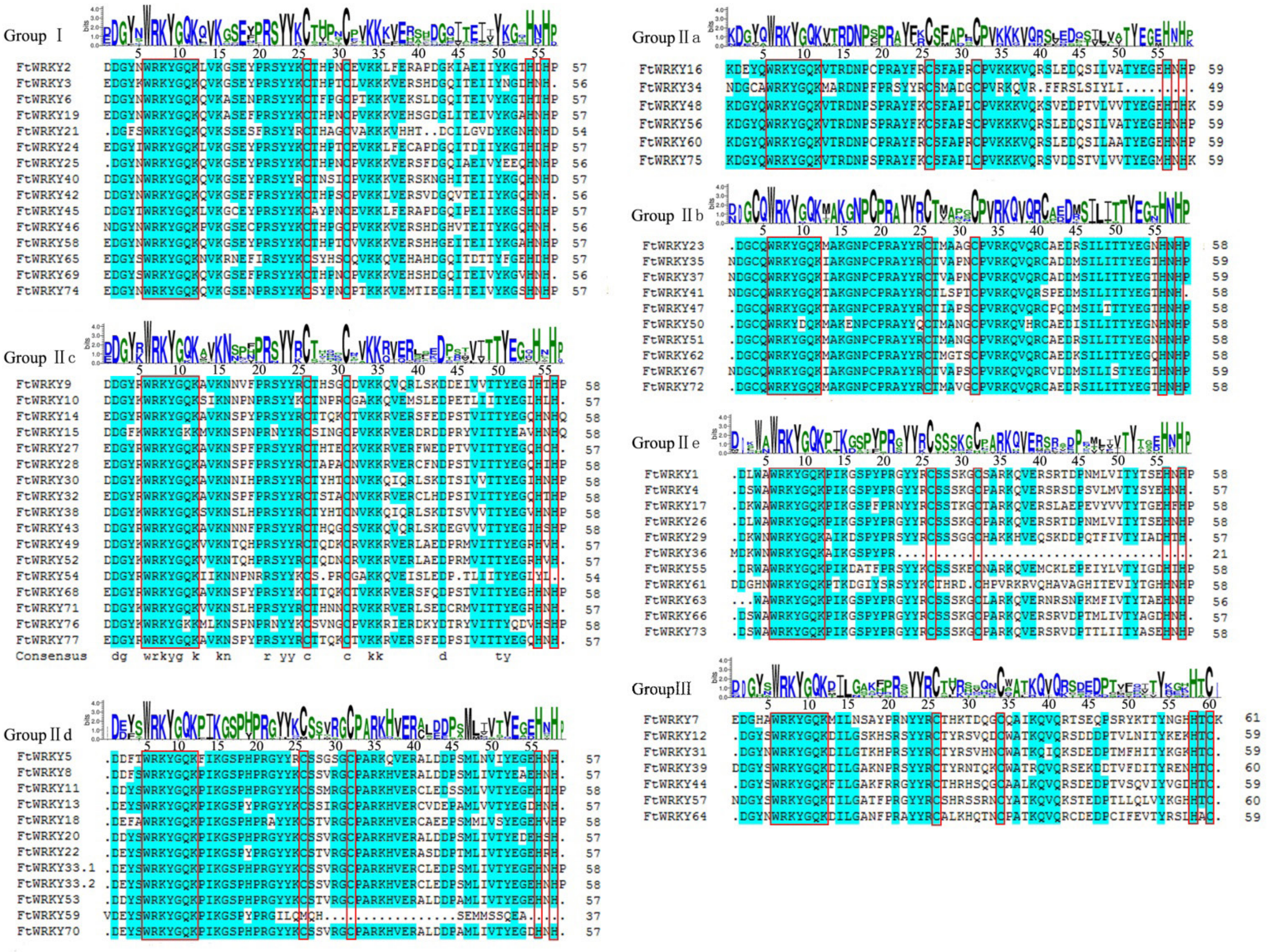

Supplementary Fig. 1. Multiple sequence alignment of the WRKY domain in Tartary buckwheat.

Supplementary Table 1. Number of WRKY genes identified in various species.

Group	*F.tatarium*	*Arabidposis thaliana*	*Oryza sativa*	*Triticum aestivum*	*Apium graveolens*	*Camellia sinensis*	*Populus trichocarpa*	*Glycine max*
I	16	32	34	25	10	13	50	32
II a	6	3	4	5	6	4	5	14
II b	10	8	8	1	8	3	9	33
II c	17	7	7	12	21	12	13	42
II d	12	8	11	12	6	7	13	21
II e	10	-	-	5	12	5	4	20
III	7	14	36	33	6	6	10	26
Total	78	72	100	93	69	50	104	188

Supplementary Table 2. The 14 representative WRKYs in *Arabidopsis thaliana* included in this study.

Name	Gene ID	Subfamily
AtWRKY6	At1g62300	II b
AtWRKY8	At5g46350	II c
AtWRKY9	At1g68150	II b
AtWRKY12	At2g44745	II c
AtWRKY14	At1g30650	II e
AtWRKY15	At2g23320	II d
AtWRKY18	At4g31800	II a
AtWRKY21	At2g30590	II d
AtWRKY25	At2g30250	I
AtWRKY33	At2g38470	I
AtWRKY40	At1g80840	II a
AtWRKY46	At2g46400	III
AtWRKY54	At2g40750	III
AtWRKY65	At1g29280	II e

Supplementary Table 3. FtWRKY primers used in the expression pattern analysis.

Gene name	Forward (5'-3')	Reverse (5'-3')
FtWRKY6	ATCCTCTGCTTCCATGGCTT	AGACCTGCTTCTCTGTGACC
FtWRKY7	CCGACGACGACAACAATCAT	TGAAACAGGAGCAAGCGAAG
FtWRKY31	CCCACGATGTTCCACATCAC	CATGGCGGAATCTTGTTGCT
FtWRKY74	ACCGGAGAGGCTGTTACAAA	ACTTGCTTGATTGCTTGGCA
Histon3	AAGGAAGCAATTGGCAAC	TCACGAAGAGCAACGGTA

Supplementary Table 4. Details of the identified motif sequences.

Motif	*E* value	Width	Site	Best possible match
1	1.0E-1685	29	76	LEDGYRWRKYGQKVVKGNPYPRSYYKCTH
2	7.0E-716	24	56	GCPVRKQVQRCAEDRSILITTYEG
3	4.80E-276	41	11	DGYNWRKYGQKQVKASEFPRSYYKCTHPNCPVKKKVEHSGD
4	1.2E-166	21	31	KRKKKEREPRIAFMTKSEVDH
5	8.5E-166	49	10	NHNHPLPPAAMAMASTTTAAASMLLSGSMPSADGLMNPNFLARTILPCS
6	8.6E-162	15	29	ITEIIYKGAHNHPKP
7	1.4E-137	21	14	GRCHCSKRRKSRVKRVVRVPA
8	5.5E-105	29	14	FLVEQMASSLTKDPTFTAALAAAISGRFM
9	1.40E-94	15	17	GCPARKHVERALDDP
10	3.70E-89	48	10	TELAKLQVELEQMNIENERLKGMLAQVSQNYTALQMHVVAIMQQHQNQ
